# Sudden cardiac arrest mortality in China: temporal trends and risk factors

**DOI:** 10.1186/s40779-025-00639-7

**Published:** 2025-08-15

**Authors:** Yang Yu, Jie Wang, Ji-Fei Wang, Jiang-Mei Liu, Xiao-Jie Liu, Yu-Chen Gao, Sudena Wang, Yao Ding, Yao Lu, Mai-Geng Zhou, Marie Ng, Hu-Shan Ao

**Affiliations:** 1 Department of Anesthesiology, Fuwai Hospital, Chinese Academy of Medical Sciences and Peking Union Medical College, Beijing, 100037 China; 2 Clinical Research Center, the Third Xiangya Hospital, Central South University, Changsha, 410013 China; 3 National Center for Chronic and Noncommunicable Disease Control and Prevention, Chinese Center for Disease Control and Prevention, Beijing, 100050 China; 4 Department of Anesthesiology, the Affiliated Hospital of Qingdao University, Qingdao, 266003 Shandong China; 5 Department of Anesthesiology, University Medical Center, Johannes Gutenberg-University Mainz, 55131 Mainz, Germany; 6 Yong Loo Lin School of Medicine, National University of Singapore, Singapore, 117599 Singapore

**Keywords:** Sudden cardiac death (SCD), Mortality rate, Age-standardized mortality rates (ASMR), National mortality surveillance system (NMSS)

## Abstract

**Background:**

Sudden cardiac death (SCD) accounts for more than half of all sudden death cases, posing a significant health burden in China. However, epidemiological data on SCD are scarce due to the lack of a central data registry and the heterogeneity of case definitions. This study aims to provide reliable estimates of the incidence and risk factors of SCD in China at the national and regional levels from 2013 to 2021, as well as the current status of prevention.

**Methods:**

The multi-cause mortality data from 2013 to 2021 were obtained from the National Mortality Surveillance System of China. Deaths related to cardiac arrest were identified. Crude and age-standardized mortality rates were calculated by time, and region. Joint point regression was applied to identify significant changes during the study period. Subgroup analyses and multilevel negative binomial analysis were performed to understand the SCD risk factors. The first-line prevention measures and their current implementation in China and developed countries were also determined from published articles.

**Results:**

From 2013 to 2021, the crude mortality rate of sudden cardiac arrest increased markedly from 8.36 deaths per 100,000 population in 2013 to 18.59 deaths per 100,000 population in 2021. There were considerable differences among regions. Subgroup analysis and negative binomial regression results indicated that males and the elderly were at higher risk of SCD. SCD may be associated with poor medical conditions. More than half of SCDs occurred outside hospitals, and approximately 60% of SCDs were related to ischemic heart disease as the underlying cause. Currently, developed countries have widely adopted primary prevention and emergency treatment measures; however, the utilization rate of such measures in China is relatively low and should be improved.

**Conclusions:**

With the continuous rise in the prevalence of cardiovascular diseases and their related risk factors in China, the burden of SCD is expected to increase. In addition to strengthening the clinical pathways for sudden cardiac arrest cases in pre-hospital and hospital settings, it is also necessary to enhance public awareness, knowledge and first-line practical training through large-scale policies for governmental and community-based projects.

**Supplementary Information:**

The online version contains supplementary material available at 10.1186/s40779-025-00639-7.

## Background

Sudden cardiac death (SCD) is a major and common clinical and public health issue globally, causing 4–5 million cases annually [1], accounting for more than half of all cardiovascular deaths worldwide [2, 3]. Even in developed countries such as the United States and European countries, over 350,000 people die from SCD each year, and resuscitation measures still need further exploration [4, 5]. In China, a previous project titled “the clinical applications of implantable cardioverter defibrillators (ICDs) and the prevention of sudden cardiac death” showed that the overall incidence rate of SCD was 41.8 per 100,000 population [6], equating to approximately 544,000 deaths per year. However, this study was based on surveys of only 4 cities in China. Another study based on a comprehensive literature review also reached a similar conclusion, estimating the incidence of SCD at 40.7 per 100,000 person-years [7]. The increasing prevalence of cardiovascular diseases (CVDs) and the large world population indicate that the incidence of SCD is on the rise [8], and premature mortality rates are also increasing [4, 9]. SCD also poses significant challenges to public health management in China [10]. However, the above epidemiological data on SCD in China are only from a few cities and lack national representativeness. Definitions of SCD vary among studies, and data sources range from disease registries to retrospective autopsies [11, 12]. Furthermore, the disease and risk factor profiles in China have undergone significant changes in recent decades [10, 13], resulting in insufficient epidemiological evidence on SCD in China and a lack of understanding of its burden, key areas, and affected populations.

The incidence of SCD is closely related to the survival outcomes of patients with cardiac arrest. In China, the survival rate of cardiac arrest is significantly lower than that in other countries [14, 15]. A prospective investigation of 12 hospitals in Beijing, China, reported that the in-hospital survival rate of cardiac arrest was approximately 9.1%, which was lower than that reported levels in other industrialized countries [16]. Out-of-hospital cardiac arrest is the main cause of SCD, with a poor prognosis and consistently low survival rate. According to the results of a large-scale national registry study, the Baseline Investigation of Out-of-hospital Cardiac Arrest (BASIC-OHCA), the burden of out-of-hospital cardiac arrest assessed by emergency medical services is heavy, and the proportion of resuscitation attempts is low [17]. A recent meta-analysis indicated that even for patients who received out-of-hospital cardiopulmonary resuscitation (CPR), the discharge survival rate was only 1.8% [15]. However, most studies have shown that timely emergency measures such as CPR can effectively reduce the mortality rate of out-of-hospital cardiac arrest [18–22]. This suggests that current preventive measures may be deficient in practice, resulting in unsatisfactory effectiveness. Thus, strategy implementation of these interventions requires not only a comprehensive understanding of temporal trends and geographic patterns of disease burden and identification of susceptible subgroups but also an understanding of the current state of preventive practice and identification of current gaps. These findings suggest that SCD can be prevented using various strategies, including improvements in bystander response and the prescription of preventative and therapeutic treatments for high-risk individuals [15].

This study utilized the National Mortality Surveillance System (NMSS), one of the most extensive central mortality surveillance databases in China, to estimate the situation of SCD in the Chinese population. It aimed to delineate the geographical and temporal variations of SCD across China, explore the sociodemographic determinants associated with SCD, and compare the current status of SCD prevention in China. The findings of this study shed light on the potential deficiencies in SCD prevention and treatment interventions in the country.

## Methods

### Study design and data sources

We conducted a population-based longitudinal analysis using mortality data extracted from the NMSS initiated in 1978 by the Chinese government. In 2013, the Chinese government combined the system with the national vital registration system, creating a data collection system from 605 surveillance points [23, 24]. The cause of death section in the standard certificate of death in China (Additional file 1: Table S1) was the same as the standard certificate of death in the United States. Data quality is reviewed annually by the quality control committee. In addition, staff training, the development of regulations for death registration, and site quality inspection are performed to ensure compliance with World Health Organization guidelines [25]. The combination of the disease point surveillance system and vital registration system allows robust estimation of cause-specific mortality at the national and regional levels. Although mortality data before 2013 are available from the former vital registration system, to maintain data consistency, our study focuses on mortality records from January 1, 2013, to December 31, 2021.

The NMSS covers 323.8 million residents, approximately 24.3% of the total population of China. Detailed information about NMSS is provided in Additional file 1: Materials and methods. For deaths that occurred in the hospital setting, the causes of death were recorded and confirmed by clinical staff. For deaths that occurred outside the hospital, verbal autopsies were conducted by community health workers, and subsequently coded and verified by local hospital staff [25]. Rigorous quality control was used at both the local and national levels to verify the accuracy and completeness of the coding. The study was centrally approved by the ethics committee of Fuwai Hospital (2023–2100).

### Procedures

SCD was identified using the International Classification of Diseases, 10th Revision (ICD-10) and textual descriptions in the death certificate (Additional file 1: Table S2). A death was SCD if one of the following criteria was met: 1) the presence of ICD codes I46 or I49.0; 2) the presence of R95 or R96 along with any I-series codes or Q20–Q28; 3) descriptions of SCD and its synonyms in clinical notes; or 4) descriptions of sudden cardiac arrest in clinical notes and the presence of any I-series code or Q20–Q28. Records were excluded if the basic cause of death involved accidental injury, malignancy, acquired immunodeficiency syndrome, syphilis, cerebrovascular disease, aging, or mental disorder-like illnesses due to substance use. Following the initial data extraction, death certificates were reviewed by two clinically experienced physicians to identify and eliminate patients with non-SCD, such as acute pulmonary embolism and airway foreign bodies (Additional file 1: Fig. S1).

Five sociodemographic factors, the place of death (medical institution, on the way to the hospital, home or nursing facility, other, or not available), and the cause of death information (ICD-10 classification based on cause of death surveillance reports) were considered in our analysis. The sociodemographic information included gender (male or female), age (0–14 years, 15–34 years, 35–64 years, and ≥ 65 years), marital status (unmarried, married, widowed, or divorced), location (urban or rural), and region (Eastern, Middle, or Western).

We also explored the current status of SCD prevention in China. Owing to the lack of national survey data concerning SCD prevention, we extracted relevant data from published literature to compare the prevalence and utilization of SCD prevention in China and developed countries. The important prevention methods for SCD management include automated external defibrillators (AEDs), ICDs, CPR, and related training.

### Statistical analysis

The 2013–2021 national total population by marital status was estimated based on 2010 and 2020 China census data. Age-standardized mortality rate (ASMR) was calculated using the census population in 2020 as a reference. Mortality rates were calculated per 100,000 person-years. In addition, the crude mortality rate was calculated and compared with ASMR to understand the influence of age structure. Joint point regression analyses were performed to examine the mortality trends of the overall population and subgroups from 2013 to 2021 and to identify substantial changes in the trends. To determine the influence of the COVID-19 pandemic on the incidence of SCD, the study periods were divided into “pre-COVID-19 period” (2013–2019) and “COVID-19 period” (2020–2021). The average annual percentage changes (AAPCs) and 95% confidence intervals (CIs) for the whole period and each subgroup period, as well as the annual percentage changes (APCs) for each different time, were calculated to quantify significant changes.

Multilevel negative binomial regression was used to investigate whether there are geographic variations across different provinces in SCD mortality, and the effect of risk factors on SCD. The number of deaths was stratified into 21,204 strata by year, gender, province, location, region, and age (5 year age groups). The number of deaths in these strata was fitted at level 1 of the multilevel model. The geographic variation between provinces was fitted at level 2 of the multilevel model. The baseline model includes variables of demographics (year, gender, location, and region). Provincial-level variables include socioeconomics and medical and health care resources (GDP per capita, number of doctors per 1000 population, number of hospital beds per 1000 population, mean years of education, and sectoral employment distribution), all in quartiles, were introduced into models. All fixed-effect parameters were exponentiated to odds ratios (*OR*s) with 95% CI.

Two-sided *Z* tests with a significance level of 0.05 were used to determine statistical significance.

Geospatial maps were created using QGIS 3.22. Joint point regression was performed using Jointpoint 4.8.0.1. Other statistical analyses were performed using SAS version 9.4.

## Results

### National SCD mortality burden in China from 2013 to 2021

**Fig. 1:**
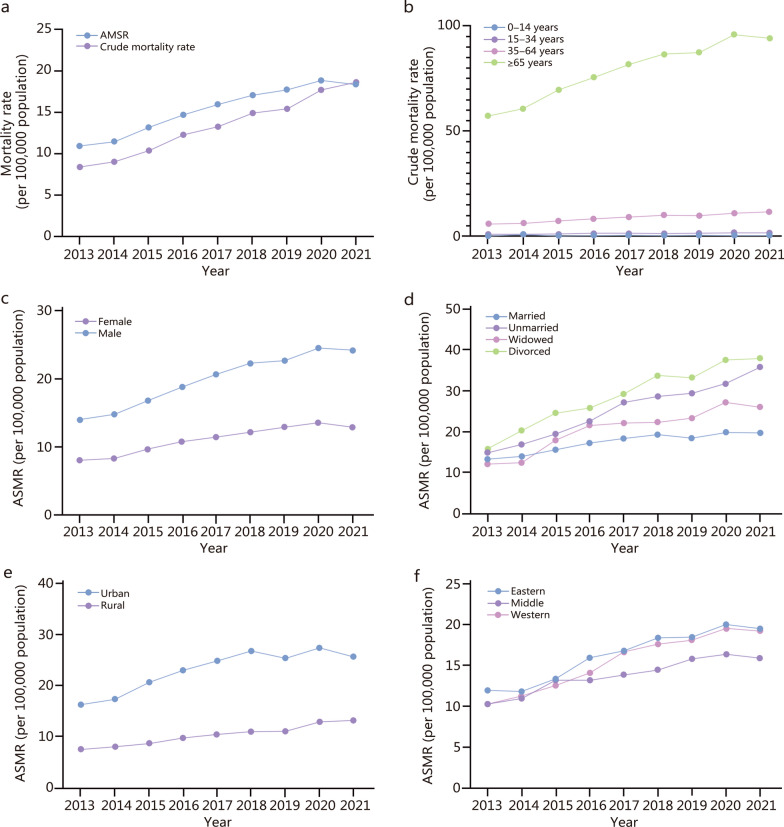
SCD mortality per 100,000 population from 2013 to 2021. **a** Total mortality. **b** Mortality by age groups. **c** ASMR by gender. **d** ASMR by marital status (married-unmarried-widowed-divorced). **e** ASMR by location (urban–rural). **f** ASMR by region (Eastern-Middle-Western). SCD sudden cardiac death, ASMR age-standardized mortality rate

Between 2013 and 2021, a total of 401,291 SCDs were recorded in the NMSS. At the national level, the estimated crude mortality rate was 8.36 deaths per 100,000 population in 2013 and steadily increased to 18.59 deaths per 100,000 population in 2021. ASMRs were considerably higher than crude mortality rates. ASMR was estimated to be 10.95 deaths per 100,000 population in 2013 and increased to 18.85 deaths per 100,000 population in 2020. This was followed by a slight but statistically non-significant decline in 2021, with a rate of 18.38 deaths per 100,000 population (Fig. 1a). Based on the crude mortality rate, the number of sudden cardiac arrest deaths was estimated to be 114,320 in 2013 and increased to 262,635 in 2021 in the country (Table 1). Upon stratification of the study periods based on the COVID-19 pandemic, our results revealed a statistically significant upward trajectory in SCD incidence during the pre-COVID-19 period (AAPC = 8.71, 95% CI 7.73–10.08). However, this upward trend appeared to plateau during the COVID-19 period, as evidenced by the absence of significant temporal variation in SCD rates (AAPC = 2.51, 95% CI –3.44 to 5.70) (Additional file 1: Table S3).

**Table 1:** National estimation of SCD-related deaths by year

Variables	2013	2014	2015	2016	2017	2018	2019	2020	2021
Numbers	114,320	124,334	143,272	170,615	185,815	208,885	217,018	249,606	262,635
Age (years, mean ± SD)	68.64 ± 17.50	68.94 ± 17.62	69.55 ± 17.32	70.12 ± 17.23	70.22 ± 17.23	70.86 ± 17.09	71.04 ± 17.05	71.56 ± 16.64	71.86 ± 16.64
Gender									
Male	71,053	77,701	88,208	104,058	114,842	128,842	133,857	153,055	161,853
Female	43,267	46,633	55,099	66,557	70,973	80,043	83,161	96,551	100,782
Location									
Urban	81,608	88,617	106,727	128,400	141,415	162,370	169,218	196,816	201,670
Rural	32,712	35,717	36,545	42,215	44,400	46,515	47,800	52,790	60,965
Region									
Eastern	55,194	57,290	66,946	81,954	88,993	102,235	101,672	119,735	122,942
Middle	32,799	35,931	44,386	46,694	49,397	53,627	58,663	64,739	69,333
Western	26,327	31,113	31,940	41,967	47,725	53,023	56,683	65,131	70,360

Region: Eastern includes Beijing, Tianjin, Hebei, Liaoning, Shanghai, Jiangsu, Zhejiang, Fujian, Shandong, Guangdong, Hainan; Middle includes Shanxi, Jilin, Heilongjiang, Anhui, Jiangxi, Henan, Hubei, Hunan; Western includes Inner Mongolia, Guangxi, Chongqing, Sichuan, Guizhou, Yunnan, Xizang, Shaanxi, Gansu, Qinghai, Ningxia, Xinjiang. *SCD* sudden cardiac death

### SCD burden based on risk factors

During the study period, negative binomial regression results demonstrated that age, gender, location, and medical and health care resources contributed to the variance among SCDs below the province scale (Table 2). Overall, 4.58% of the variation in SCD mortality between provinces was explained by the fitted variables (Table 2: Model 5). The mortality rates associated with SCD increased with age, with the highest rates observed in the ≥ 65 years group. In 2021, the mortality rate at the age of ≥ 65 years was estimated to be 94.12 per 100,000 population, which was 8.07-times higher than that of the 35–64 years group (Fig. 1b). In these 4 age groups, the AAPC is estimated to be 6.80 (95% CI 4.99–8.64) in the ≥ 65 years group, 8.87 (95% CI 6.41–11.38) in the 35–64 years group, 7.73 (95% CI 6.21–9.27) in the 15–34 years group, and 0.63 (–6.48 to 8.29) in the 0–14 years group (Additional file 1: Table S4).

**Table 2:** Associated factors of sudden cardiac mortality from NMSS in China, 2013–2021: estimated from negative binomial regression

Variables	Model 1	Model 2	Model 3	Model 4	Model 5
Fixed effects					
Year	1.065 (1.060–1.071)^*^	1.069 (1.061–1.077)^*^	1.088 (1.074–1.103)^*^	1.089 (1.075–1.104)^*^	1.092 (1.073–1.111)^*^
Age (5 year group)	1.421 (1.416–1.426)^*^	1.421 (1.416–1.426)^*^	1.421 (1.416–1.426)^*^	1.421 (1.416–1.426)^*^	1.421 (1.416–1.426)^*^
Gender					
Female	1	1	1	1	1
Male	1.930 (1.877–1.985)^*^	1.931 (1.877–1.986)^*^	1.931 (1.878–1.986)^*^	1.931 (1.878–1.986)^*^	1.931 (1.877–1.986)^*^
Location					
Rural	1	1	1	1	1
Urban	1.900 (1.846–1.956)^*^	1.902 (1.847–1.957)^*^	1.903 (1.849–1.959)^*^	1.903 (1.849–1.959)^*^	1.904 (1.850–1.960)^*^
Region					
Eastern	1	1	1	1	1
Middle	1.002 (0.597–1.680)	0.965 (0.582–1.602)	0.947 (0.567–1.581)	0.945 (0.564–1.581)	0.886 (0.530–1.481)
Western	1.017 (0.639–1.619)	0.965 (0.610–1.527)	0.966 (0.607–1.538)	0.938 (0.586–1.500)	0.846 (0.523–1.371)
GDP per capita					
< 11,895		1	1	1	1
11,895–21,237.1		1.166 (1.084–1.255)^*^	1.173 (1.089–1.265)^*^	1.184 (1.097–1.276)^*^	1.186 (1.100–1.279)^*^
21,237.1–36,013.8		1.046 (0.940–1.165)	1.053 (0.942–1.176)	1.053 (0.943–1.177)	1.058 (0.946–1.183)
> 36,013.8		0.961 (0.838–1.102)	0.962 (0.835–1.109)	0.959 (0.832–1.106)	0.965 (0.836–1.115)
Number of doctors per 1000 population					
< 57			1	1	1
57–65			0.992 (0.939–1.048)	0.985 (0.932–1.041)	0.994 (0.939–1.051)
65–74			0.966 (0.898–1.039)	0.958 (0.890–1.031)	0.970 (0.899–1.046)
74			0.869 (0.793–0.952)^*^	0.865 (0.788–0.948)^*^	0.882 (0.801–0.971)^*^
Number of hospital beds per 1000 population					
< 48.88			1	1	1
48.88–55.92			0.981 (0.925–1.041)	0.977 (0.921–1.037)	0.983 (0.926–1.043)
55.92–64.08			0.980 (0.906–1.061)	0.980 (0.905–1.060)	0.988 (0.912–1.070)
> 64.08			0.952 (0.857–1.057)	0.948 (0.854–1.053)	0.957 (0.861–1.064)
Mean years of education					
< 8.76				1	1
8.76–9.20				0.926 (0.861–0.995)^*^	0.925(0.860–0.995)^*^
9.20–9.56				0.945 (0.868–1.029)	0.933 (0.855–1.018)
> 9.56				0.961 (0.867–1.066)	0.957 (0.862–1.062)
Sectoral employment distribution					
The proportion of employment in the primary sector					1.054 (0.040–27.704)
The proportion of employment in the secondary sector					0.637 (0.025–16.151)
The proportion of employment in the tertiary sector					0.689 (0.026–18.353)
Random effects					
Variance among provinces (SE)	0.320 (0.087)	0.307 (0.084)	0.313 (0.086)	0.316 (0.087)	0.306 (0.087)
MRR (provinces)	1.714	1.696	1.707	1.710	1.696
PCV (provinces) (%)	–	4.12	2.37	1.41	4.58

Data for fixed effects are presented in odds ratio and 95% confidence intervals. Region: Eastern includes Beijing, Tianjin, Hebei, Liaoning, Shanghai, Jiangsu, Zhejiang, Fujian, Shandong, Guangdong, Hainan; Middle includes Shanxi, Jilin, Heilongjiang, Anhui, Jiangxi, Henan, Hubei, Hunan; Western includes Inner Mongolia, Guangxi, Chongqing, Sichuan, Guizhou, Yunnan, Xizang, Shaanxi, Gansu, Qinghai, Ningxia, Xinjiang. ^*^P < 0.05. “–” indicates no data. *MRR* median rate ratio (an MRR of 1 suggests no geographic variation in the outcome variable, whereas MMR > 1 indicates significant influence of geographical variables on the outcome), *PCV* proportional change in variance

As shown in Fig. 1c, considerable gender differences were observed. Across the study period, ASMR was 1.73 to 1.88-times higher in males than in females (*Z* = 57.53, *P* < 0.001). Mortality rates steadily increased for both males and females (ASMR: 14.03 deaths per 100,000 population in 2013 to 24.49 deaths per 100,000 population in 2020 among males, and from 8.03 deaths per 100,000 population in 2013 to 13.57 deaths per 100,000 population in 2020 among females). This translates to an AAPC of 7.42 (95% CI 5.59–9.29) for males and 6.30 (95% CI 3.59–9.07) for females (Additional file 1: Table S4). A slight but non-significant decline was observed in 2021, with ASMR reduced to 24.19 deaths per 100,000 population in males and 12.89 deaths per 100,000 population in females.

ASMR varies across marital statuses. The ASMR was the highest among divorced individuals and the lowest among married individuals (*Z* = 11.82, *P* < 0.001). In 2021, ASMR was estimated to be 38.24 deaths per 100,000 population among divorced individuals compared with 19.62 deaths per 100,000 population among married individuals, 35.73 deaths per 100,000 population among unmarried individuals, and 25.93 deaths per 100,000 population among widowed individuals (Fig. 1d). Except for married individuals, all other marital status subgroups presented increasing trends, with the most significant increase among divorced individuals with an AAPC of 12.21 (95% CI 7.85–16.75), followed by unmarried individuals with an AAPC of 11.45 (95% CI 9.83–13.10) (Additional file 1: Table S4).

Compared with those in rural areas, ASMR in urban areas was 1.94 to 2.45-times higher from 2013 to 2021 (*Z* = 79.04, *P* < 0.001). ASMR in urban areas increased from 16.26 deaths per 100,000 population in 2013 to 25.50 deaths per 100,000 population in 2021 (Fig. 1e). This corresponded to an estimated AAPC of 6.45 (95% CI 3.80–9.16) (Additional file 1: Table S4). ASMR in rural areas has also increased but at a slightly slower pace, with an estimated AAPC of 7.39 (95% CI 6.37–8.41) (Additional file 1: Table S4). The ASMR was estimated to be 7.54 deaths per 100,000 population in 2013 and 13.16 deaths per 100,000 population in 2021 (Fig. 1e).

Figure 1f shows the geographical differences among 3 regions in China. In general, the ASMR was significantly higher in the Eastern region than in the Western (*Z* = 5.50, *P* < 0.001) and Middle regions (*Z* = 16.31,* P* < 0.001). In 2021, the estimated mortality rates were 19.51 deaths per 100,000 population, 15.88 deaths per 100,000 population, and 19.22 deaths per 100,000 population in the Eastern, Middle, and Western regions, respectively. The Western region (AAPC = 8.53, 95% CI 6.57–10.52) showed the most rapid increase (Additional file 1: Table S4).

**Fig. 2:**
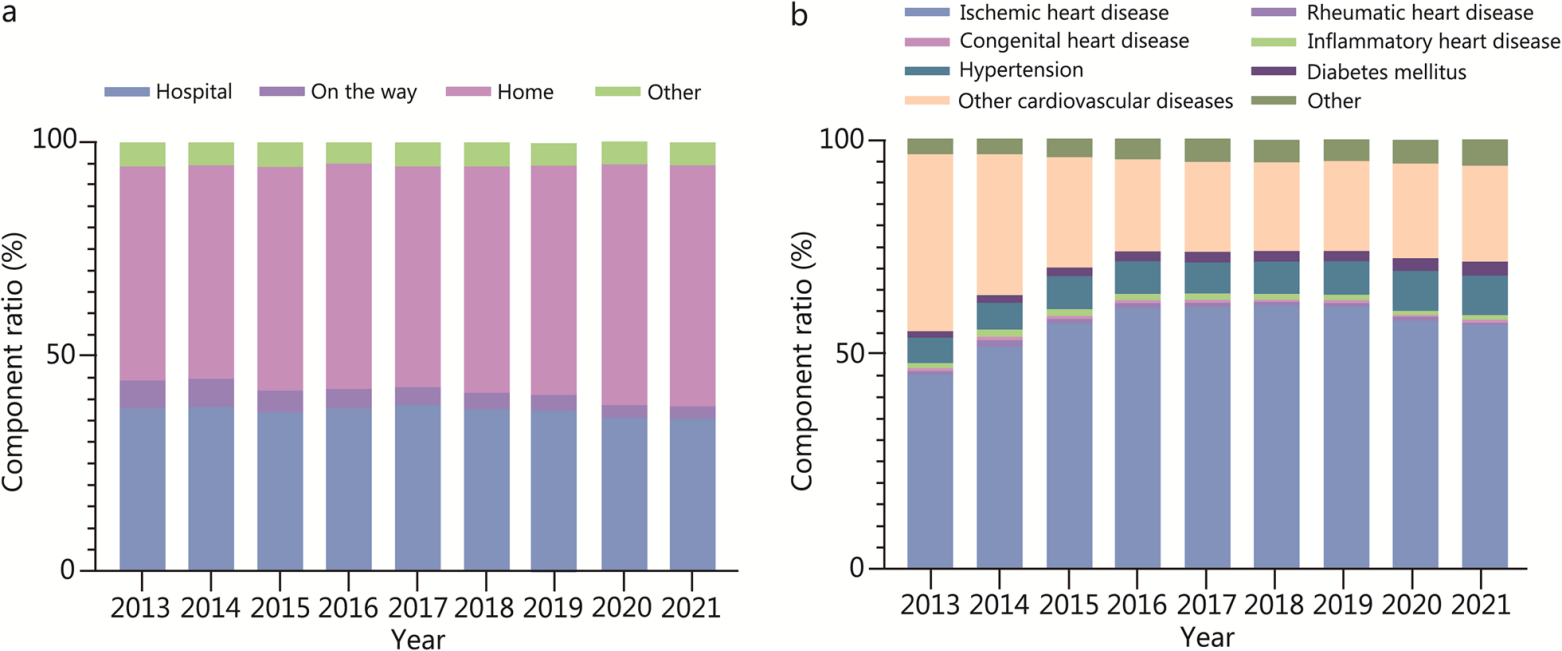
Proportion of SCD mortality from 2013 to 2021 by (**a**) place of death and (**b**) basic cause of death. SCD sudden cardiac death

As shown in Fig. 2a, approximately half of SCD cases occurred at home, and the proportion remained stable between 2013 and 2021. The proportion of SCDs that occurred in hospitals was between 35.00% and 39.00% across the 9 year study period. In terms of the basic causes of death associated with SCDs, ischemic heart disease, other CVDs, and hypertension were the top 3 causes, accounting for 56.74%, 22.32%, and 9.12% of SCD cases, respectively, in 2021 (Fig. 2b).

### Prevention practices of SCD

The current status of the trends, outcomes of sudden cardiac arrest, and important SCD prevention measures in China and developed countries are summarized in Additional file 1: Table S5. There are 4 important measures for preventing SCD, including ICDs, AEDs, CPR, and training. According to the previous surveys, the number of ICD implantations, AEDs, and use of CPR have become increasingly available, while their utilization rates and related training programs still require significant improvement [10, 17, 18, 26–31] (Additional file 1: Table S5). The training on CPR and basic life support in China currently also requires improvement [5, 29, 32, 33], and with a need for more high-standard training, as well as co-construction efforts. In China, it is necessary to further improve relevant measures to enhance public accessibility, strengthen related training, and continuously promote localization in line with national conditions.

## Discussion

This is a study to provide epidemiological data on SCD in China at the national level using data from the NMSS, an enhanced integrated disease surveillance and vital registration system. In this study, we found a steady increase in SCD mortality rates from 2013 to 2020 and a modest non-significant decrease in 2021. Substantial geographical disparities were observed, with mortality rates in 2021 ranging from 15.88 deaths per 100,000 population to 19.51 deaths per 100,000 population across regions and Western region showed the most rapid increase. Moreover, SCD mortality rates increased with age and were higher among males, urban residents, those who died of ischemic heart disease, and those who experienced sudden cardiac arrest outside of a hospital setting. We also found low utilization of SCD prevention, which may partly explain why SCD has not declined in China. Our study highlights the burden of disease in patients with SCD and provides evidence for the government to develop appropriate preventive policies.

Despite a growing awareness and advancements in sudden cardiac arrest prevention and treatment [10, 34], the number of SCDs has continued to rise in China over the past decade. For Europeans, no significant variability in the yearly incidence rates of SCD was observed [35]. Although the increase in China is partly due to improved diagnostic capabilities, this is more likely due to the continuous increase in CVD burden and the changes in risk factors and the disease spectrum in the country [36]. In the past decade, CVD has become the first major cause of death in China [37]. In 2019, CVD accounted for 46.74% and 44.26% of all deaths in rural and urban areas, respectively [38]. Despite a moderate reduction, the prevalence of smoking in China remains high [39], whereas the prevalence of obesity and hypertension is increasing [40]. Without effective strategies to prevent and manage CVDs and their associated risk factors, a high SCD mortality rate will likely persist. Our study revealed a minor decline in SCD mortality rates in 2021. It is unclear whether this is driven by an actual downward trend or is a result of data anomalies stemming from COVID-19 [41–43]. This is because standard medical coding practices were disrupted early in the pandemic, resulting in gaps in cause-of-death data [44].

At the individual level, SCD mortality rates differ based on sociodemographic characteristics, including age, gender, and marital status. Elderly individuals and males are prone to SCD at a relatively high rate, which can be attributed to their severe risk factors. With respect to marital status, the lowest mortality rates were observed among married individuals. This finding aligns with those of previous studies, suggesting that marriage potentially enhances financial and psychological stability [45] and serves as a protective factor against mortality [46]. In contrast, divorced and unmarried individuals tended to live alone and were more prone to isolation and poorer health outcomes [47, 48]. It is essential to expand social services to address the unmet needs of this susceptible population to provide more timely emergency care for the elderly and singles.

At the area level, significant regional disparities in SCD mortality rates reveal underlying differences in epidemiological profiles and socioeconomic development. Driven by variability in CVD incidence and associated risk factors [49], ASMR is different in regions with higher in Eastern region. Moreover, we observed a higher incidence of SCD in urban areas, which may be attributed to the generally elevated levels of air pollution, unfavorable temperatures, and roadway proximity in these regions [50–52]. Additionally, people living in urban areas are more likely to experience psychological stress [53], which could also contribute to elevated SCD risk, particularly sudden unexplained cardiac death [54, 55]. Although the incidence rates are higher in these developed regions, it must be acknowledged that their public prevention measures are better equipped. For example, Shanghai and Zhejiang are among the areas with the highest per capita ICD implementation [10].

The majority of SCD cases occur outside of hospital settings, and the survival rate of out-of-hospital cardiac arrest in China is low, remaining at approximately 2% [15–17, 56, 57]. Several contributing factors, including insufficient primary prevention, poor public knowledge, and awareness of proper responses during sudden cardiac events and capacity constraints in existing prehospital emergency systems, have been reported [14, 58]. Our research results also indicated that the accessibility of medical resources is an important factor affecting SCD (Table 2). Notably, ischemic heart disease, the leading cause of SCD, is currently effectively managed in clinical practice. Owing to the increased utilization of reperfusion therapies such as percutaneous coronary intervention and coronary artery bypass grafting, acute myocardial infarction patients exhibited favorable outcomes [59, 60]. In addition, for other high-risk SCD patients with comorbid CVDs, valve surgeries play an essential role in preventing SCD by restoring normal valve function and improving cardiac hemodynamics. In China, although the application of these surgeries has increased, the prevention and treatment of SCD after revascularization remain relatively inadequate at present. ICDs have become important procedures for both primary and secondary prevention of SCD. However, despite their proven efficacy [61, 62], ICD utilization remains suboptimal worldwide. In China, the implantation rates of ICD are substantially low [26, 63]. Even in the United States, underutilization persists among high-risk populations. However, although ICD therapy is critical for prevention, it can only address a small proportion of SCD cases.

In fact, the majority of SCD events occur in the general population—predominantly among individuals without prior clinical diagnosis of cardiac disease. This underscores the importance of public health initiatives to make AEDs more accessible. AEDs education and availability have greatly changed our approach to reviving out-of-hospital cardiac arrest patients regardless of their pre-existing health status and currently form the cornerstone of CPR and advanced life support protocols [19]. Regrettably, the coverage of AEDs is generally low in some areas in China, and the placement of AEDs lacks strategic planning [27, 29, 64, 65]. Although progress has been made in recent years in some provinces, such as Hangzhou, Shanghai, Shenzhen, and Haikou, the implementation of SCD prevention policies remains in the early phases and is not optimal in other regions of China [27, 66]. In 2016, the 525+ project was launched to promote CPR training and awareness. Since its inception, 177 training centers have been established, showing notable progress. However, uncertainty remains regarding the validation of training, indicating a need for further efforts in emergency education and practice [29, 31]. To improve the survival rate and reduce SCD, multisectoral reform is needed to improve pre- and post-hospital service connections, bystander laws, bystander training, and public knowledge [32, 33].

In addition to public health and policy interventions, cardiac arrest and SCD registries have been shown to be crucial in improving population-level outcomes. By providing timely data for epidemiological analysis, sudden cardiac arrest registries highlight gaps in care linkages among hospital, forensic and ambulance services. In China, the establishment of sudden cardiac arrest registries remains at the naissance stage. Efforts in nationwide data collection have recently started. Two registration systems, Baseline Investigation of In-hospital Cardiac Arrest (BASIC-IHCA) [67] and BASIC-OHCA [66], have been established to capture in-hospital cardiac arrest and out-of-hospital cardiac arrest, respectively. As the coverage of registries continues to expand, they will provide invaluable and confident insights for monitoring SCD burden and serve as a source of cross-validation for existing national disease and mortality surveillance programs.

To our knowledge, this was a study to demonstrate the temporal trends and risk factors of SCD in China at the national level. One strength was that our data were derived from the NMSS, which was designed to achieve national representativeness. Moreover, we also performed stratified analyses to elucidate SCD. This study also has several limitations. First, accurate ascertainment of causes of death is challenging, as a previous study suggested that SCD inferred from death certificates is prone to overestimation [13]. In particular, a large proportion of cardiac arrests were reported outside of hospitals, making it difficult to verify the actual cause of death and introducing potential errors. Second, we did not distinguish between the actual cause of death and the mechanism of death. Cardiac arrest is a common mechanism of death, but may not be the primary cause [67]. In the absence of a complete medical history or autopsies, confirming actual causes is impossible. Third, owing to the lack of relevant data at the individual level, it was not possible to perform an analysis to quantify the effects of disease-specific risk factors such as obesity, dyslipidemia, COVID-19, and pre-hospital emergency care. Although the results of AAPC stratified based on pre-COVID-19 periods and COVID-19 periods revealed differences in SCD incidence trends, causal attribution remains inconclusive. Future longitudinal studies with robust designs are essential to clarify the relationship between the COVID-19 pandemic and SCD. Fourth, data related to SCD prevention and control measures are lacking, so we are unable to estimate the utilization rate at the national level. Future studies should systematically document SCD onsets and emergency response data to strengthen prevention efforts. Nevertheless, we contend that our current estimates, despite the inherent constraints, address the important data gaps in the literature by offering insights into geographical and temporal trends of SCD.

## Conclusions

In conclusion, SCD is a major public health threat in China. There is an urgent need to intensify existing interventions, including strengthening public knowledge and awareness of emergency response and expanding AEDs placement, CPR and basic life support training. To improve progress in the treatment of cardiac arrest and increase the survival rate, efforts should be made at the national level. It is also essential to strengthen the implementation of public emergency measures such as AEDs and provide training for the public.

## Supplementary Information

**Additional file 1. Materials and methods. Table S1** Standard certificate of death in China_English edition. **Table S2** Codes used for screening in International Statistical Classification of Diseases and Related Health Problems 10th Revision ICD-10)-2015-World Health Organization version. **Table S3** Average annual percent change (AAPC) of sudden cardiac mortality by periods in China. **Table S4** Average annual percent change (AAPC) of sudden cardiac mortality stratified by sociodemographic factors in China. **Table S5** Comparison of current status of trends, outcomes, and prevention practices for sudden cardiac arrest. **Fig. S1** Flowchart for screening population for sudden cardiac death in China from 2013 to 2021

## Data Availability

Data cannot be made available a priori for institutional policy but can be provided upon reasonable request.

## Abbreviations

AAPC: Average annual percentage change
AEDs: Automated external defibrillators
BASIC-OHCA: Baseline investigation of out-of-hospital cardiac arrest
CIs: Confidence intervals
CPR: Cardiopulmonary resuscitation
ICDs: Implantable cardioverter defibrillators
ICD-10: International classification of diseases, 10th revision
NMSS: National mortality surveillance system
OR: Odds ratio
SCD: Sudden cardiac death
ASMR: Age-standardized mortality rate

## References

[1] Chugh SS, Reinier K, Teodorescu C, Evanado A, Kehr E, Al Samara M, et al. Epidemiology of sudden cardiac death: clinical and research implications. Prog Cardiovasc Dis. 2008;51(3):213–28. 19026856 10.1016/j.pcad.2008.06.003 PMC2621010

[2] Krokhaleva Y, Vaseghi M. Update on prevention and treatment of sudden cardiac arrest. Trends Cardiovasc Med. 2019;29(7):394–400. 30449537 10.1016/j.tcm.2018.11.002 PMC6685756

[3] Zipes DP, Camm AJ, Borggrefe M, Buxton AE, Chaitman B, Fromer M, et al. ACC/AHA/ESC 2006 guidelines for management of patients with ventricular arrhythmias and the prevention of sudden cardiac death: a report of the American college of cardiology/American heart association task force and the European society of cardiology committee for practice guidelines (writing committee to develop guidelines for management of patients with ventricular arrhythmias and the prevention of sudden cardiac death). J Am Coll Cardiol. 2006;48(5):e247–346. 16949478 10.1016/j.jacc.2006.07.010

[4] Lippert FK, Raffay V, Georgiou M, Steen PA, Bossaert L. European Resuscitation Council guidelines for resuscitation 2010 section 10. The ethics of resuscitation and end-of-life decisions. Resuscitation. 2010;81(10):1445–51 10.1016/j.resuscitation.2010.08.013 20956043

[5] Girotra S, Dukes KC, Sperling J, Kennedy K, Del Rios M, Crowe R, et al. Emergency medical service agency practices and cardiac arrest survival. JAMA Cardiol. 2024;9(8):683–91. 38837166 10.1001/jamacardio.2024.1189 PMC11154368

[6] Hua W, Zhang LF, Wu YF, Liu XQ, Guo DS, Zhou HL, et al. Incidence of sudden cardiac death in China: analysis of 4 regional populations. J Am Coll Cardiol. 2009;54(12):1110–8. 19744622 10.1016/j.jacc.2009.06.016

[7] Feng XF, Hai JJ, Ma Y, Wang ZQ, Tse HF. Sudden cardiac death in mainland China: a systematic analysis. Circ Arrhythm Electrophysiol. 2018;11(11):e006684. 30571181 10.1161/CIRCEP.118.006684

[8] GBD 2019 Diseases and Injuries Collaborators. Global burden of 369 diseases and injuries in 204 countries and territories, 1990–2019: a systematic analysis for the global burden of disease study 2019. Lancet. 2020;396(10258):1204–22. 33069326 10.1016/S0140-6736(20)30925-9 PMC7567026

[9] Kong MH, Fonarow GC, Peterson ED, Curtis AB, Hernandez AF, Sanders GD, et al. Systematic review of the incidence of sudden cardiac death in the United States. J Am Coll Cardiol. 2011;57(7):794–801. 21310315 10.1016/j.jacc.2010.09.064 PMC3612019

[10] Zhang S. Sudden cardiac death in China: current status and future perspectives. Europace. 2015;17(Suppl 2):ii14–8. 26842111 10.1093/europace/euv143

[11] Ding Z, Yang M, Wang Y, Wu S, Qiu X, Liu Q. Retrospective analysis of 769 cases of sudden cardiac death from 2006 to 2015: a forensic experience in China. Forensic Sci Med Pathol. 2017;13(3):336–41. 28752200 10.1007/s12024-017-9888-z

[12] Wang H, Yao Q, Zhu S, Zhang G, Wang Z, Li Z, et al. The autopsy study of 553 cases of sudden cardiac death in Chinese adults. Heart Vessels. 2014;29(4):486–95. 23836068 10.1007/s00380-013-0388-0

[13] Adabag AS, Luepker RV, Roger VL, Gersh BJ. Sudden cardiac death: epidemiology and risk factors. Nat Rev Cardiol. 2010;7(4):216–25. 20142817 10.1038/nrcardio.2010.3 PMC5014372

[14] Wang J, He Y, Chen X, Chen M, Tang C, Lu F, et al. A retrospective study on epidemiological analysis of pre-hospital emergency care in Hangzhou, China. PLoS ONE. 2023;18(4):e0282870. 37071636 10.1371/journal.pone.0282870 PMC10112809

[15] Zhou G, Wang Y, Sun Z, Yuan M, Ma Y, Wu Q, et al. Survival outcome among patients with out-of-hospital cardiac arrest who received cardiopulmonary resuscitation in China: a systematic review and meta-analysis. Eur J Med Res. 2023;28(1):8. 36600249 10.1186/s40001-022-00955-x PMC9811716

[16] Shao F, Li CS, Liang LR, Qin J, Ding N, Fu Y, et al. Incidence and outcome of adult in-hospital cardiac arrest in Beijing, China. Resuscitation. 2016;102:51–6. 26924514 10.1016/j.resuscitation.2016.02.002

[17] Zheng J, Lv C, Zheng W, Zhang G, Tan H, Ma Y, et al. Incidence, process of care, and outcomes of out-of-hospital cardiac arrest in China: a prospective study of the BASIC-OHCA registry. Lancet Public Health. 2023;8(12):e923–32. 37722403 10.1016/S2468-2667(23)00173-1

[18] Kitamura T, Kiyohara K, Sakai T, Matsuyama T, Hatakeyama T, Shimamoto T, et al. Public-access defibrillation and out-of-hospital cardiac arrest in Japan. N Engl J Med. 2016;375(17):1649–59. 27783922 10.1056/NEJMsa1600011

[19] Elhussain MO, Ahmed FK, Mustafa NM, Mohammed DO, Mahgoub IM, Alnaeim NA, et al. The role of automated external defibrillator use in the out-of-hospital cardiac arrest survival rate and outcome: a systematic review. Cureus. 2023;15(10):e47721. 38021997 10.7759/cureus.47721 PMC10676231

[20] Hasselqvist-Ax I, Riva G, Herlitz J, Rosenqvist M, Hollenberg J, Nordberg P, et al. Early cardiopulmonary resuscitation in out-of-hospital cardiac arrest. N Engl J Med. 2015;372(24):2307–15. 26061835 10.1056/NEJMoa1405796

[21] Sasson C, Rogers MA, Dahl J, Kellermann AL. Predictors of survival from out-of-hospital cardiac arrest: a systematic review and meta-analysis. Circ Cardiovasc Qual Outcomes. 2010;3(1):63–81. 20123673 10.1161/CIRCOUTCOMES.109.889576

[22] El-Zein RS, Kennedy KF, Chan PS. Out-of-hospital cardiac arrest survival when CPR is initiated by first responders. Resuscitation. 2023;190:109914. 37506814 10.1016/j.resuscitation.2023.109914 PMC10529146

[23] Liu S, Wu X, Lopez AD, Wang L, Cai Y, Page A, et al. An integrated national mortality surveillance system for death registration and mortality surveillance, China. Bull World Health Organ. 2016;94(1):46–57. 26769996 10.2471/BLT.15.153148 PMC4709796

[24] Wang L, Wu Y, Yin P, Cheng P, Liu Y, Schwebel DC, et al. Poisoning deaths in China, 2006–2016. Bull World Health Organ. 2018;96(5):314–26. 29875516 10.2471/BLT.17.203943 PMC5985423

[25] Yang G, Hu J, Rao KQ, Ma J, Rao C, Lopez AD. Mortality registration and surveillance in China: history, current situation and challenges. Popul Health Metr. 2005;3(1):3. 15769298 10.1186/1478-7954-3-3 PMC555951

[26] Mond HG, Proclemer A. The 11th world survey of cardiac pacing and implantable cardioverter-defibrillators: calendar year 2009–a World Society of Arrhythmia’s project. Pacing Clin Electrophysiol. 2011;34(8):1013–27. 21707667 10.1111/j.1540-8159.2011.03150.x

[27] Zhang L, Li B, Zhao X, Zhang Y, Deng Y, Zhao A, et al. Public access of automated external defibrillators in a metropolitan city of China. Resuscitation. 2019;140:120–6. 31129230 10.1016/j.resuscitation.2019.05.015

[28] Rea T, Blackwood J, Damon S, Phelps R, Eisenberg M. A link between emergency dispatch and public access AEDs: potential implications for early defibrillation. Resuscitation. 2011;82(8):995–8. 21570169 10.1016/j.resuscitation.2011.04.011

[29] Xu F, Zhang Y, Chen Y. Cardiopulmonary resuscitation training in China: current situation and future development. JAMA Cardiol. 2017;2(5):469–70. 28297007 10.1001/jamacardio.2017.0035

[30] Gu XM, Li ZH, He ZJ, Zhao ZW, Liu SQ. A meta-analysis of the success rates of heartbeat restoration within the platinum 10 min among outpatients suffering from sudden cardiac arrest in China. Mil Med Res. 2016;3: 6. 27006782 10.1186/s40779-016-0071-8 PMC4802919

[31] Shao F, Li CS, Liang LR, Li D, Ma SK. Outcome of out-of-hospital cardiac arrests in Beijing, China. Resuscitation. 2014;85(11):1411–7. 25151546 10.1016/j.resuscitation.2014.08.008

[32] Ng TP, Eng SW, Ting JXR, Bok C, Tay GYH, Kong SYJ, et al. Global prevalence of basic life support training: a systematic review and meta-analysis. Resuscitation. 2023;186:109771. 36934835 10.1016/j.resuscitation.2023.109771

[33] Dong X, Zhang L, Wang Z, Zheng ZJ. Implementation of basic life support education for the lay public in China: barriers, enablers, and possible solutions. Front Public Health. 2024;12:1390819. 38993705 10.3389/fpubh.2024.1390819 PMC11236690

[34] Chan NY. Sudden cardiac death in Asia and China: are we different? J Am Coll Cardiol. 2016;67(5):590–2. 26846954 10.1016/j.jacc.2015.12.011

[35] Empana JP, Lerner I, Valentin E, Folke F, Böttiger B, Gislason G, et al. Incidence of sudden cardiac death in the European Union. J Am Coll Cardiol. 2022;79(18):1818–27. 35512862 10.1016/j.jacc.2022.02.041

[36] Hayashi M, Shimizu W, Albert CM. The spectrum of epidemiology underlying sudden cardiac death. Circ Res. 2015;116(12):1887–906. 26044246 10.1161/CIRCRESAHA.116.304521 PMC4929621

[37] Zhang J, Tong H, Jiang L, Zhang Y, Hu J. Trends and disparities in China’s cardiovascular disease burden from 1990 to 2019. Nutr Metab Cardiovasc Dis. 2023;33(12):2344–54. 37596135 10.1016/j.numecd.2023.07.039

[38] Center for Cardiovascular Diseases the Writing Committee of the Report on Cardiovascular Health and Diseases in China. Report on cardiovascular health and diseases in China 2021: an updated summary. Biomed Environ Sci. 2022;35(7):573–603. 10.3967/bes2024.162 39401992

[39] Spatial, temporal, and demographic patterns in prevalence of smoking tobacco use and attributable disease burden in 204 countries and territories, 1990–2019: a systematic analysis from the Global Burden of Disease Study 2019. Lancet. 2021;397(10292):2337–60. 10.1016/S0140-6736(21)01169-7 PMC8223261 34051883

[40] Afshin A, Forouzanfar MH, Reitsma MB, Sur P, Estep K, Lee A, et al. Health effects of overweight and obesity in 195 countries over 25 years. N Engl J Med. 2017;377(1):13–27. 28604169 10.1056/NEJMoa1614362 PMC5477817

[41] Lyu T, Khan FA, Sajeed SM, Kansal A, Kansal MG, Dhanvijay S, et al. In-hospital cardiac arrest incidence and outcomes in the era of COVID-19: an observational study in a Singapore hospital. Int J Emerg Med. 2021;14(1):33. 34058983 10.1186/s12245-021-00356-7 PMC8165958

[42] Bharmal M, Digrande K, Patel A, Shavelle DM, Bosson N. Impact of coronavirus disease 2019 pandemic on cardiac arrest and emergency care. Heart Fail Clin. 2023;19(2):231–40. 36863815 10.1016/j.hfc.2022.08.009 PMC9973546

[43] Chan PS, Girotra S, Tang Y, Al-Araji R, Nallamothu BK, Mcnally B. Outcomes for out-of-hospital cardiac arrest in the United States during the coronavirus disease 2019 pandemic. JAMA Cardiol. 2021;6(3):296–303. 33188678 10.1001/jamacardio.2020.6210 PMC7666759

[44] Gill JR, Dejoseph ME. The importance of proper death certification during the COVID-19 pandemic. JAMA. 2020;324(1):27–8. 32520302 10.1001/jama.2020.9536

[45] Espinosa J, Evans WN. Heightened mortality after the death of a spouse: marriage protection or marriage selection? J Health Econ. 2008;27(5):1326–42. 18513810 10.1016/j.jhealeco.2008.04.001

[46] Jaffe DH, Manor O, Eisenbach Z, Neumark YD. The protective effect of marriage on mortality in a dynamic society. Ann Epidemiol. 2007;17(7):540–7. 17434751 10.1016/j.annepidem.2006.12.006

[47] Gan T, Yang J, Jiang L, Gao Y. Living alone and cardiovascular outcomes: a meta-analysis of 11 cohort studies. Psychol Health Med. 2023;28(3):719–31. 34477038 10.1080/13548506.2021.1975784

[48] Zhao Y, Guyatt G, Gao Y, Hao Q, Abdullah R, Basmaji J, et al. Living alone and all-cause mortality in community-dwelling adults: a systematic review and meta-analysis. EClinicalMedicine. 2022;54:101677. 36204005 10.1016/j.eclinm.2022.101677 PMC9530481

[49] Zhou M, Wang H, Zeng X, Yin P, Zhu J, Chen W, et al. Mortality, morbidity, and risk factors in China and its provinces, 1990–2017: a systematic analysis for the global burden of disease study 2017. Lancet. 2019;394(10204):1145–58. 31248666 10.1016/S0140-6736(19)30427-1 PMC6891889

[50] Borchert W, Grady ST, Chen J, Deville NV, Roscoe C, Chen F, et al. Air pollution and temperature: a systematic review of ubiquitous environmental exposures and sudden cardiac death. Curr Environ Health Rep. 2023;10(4):490–500. 37845484 10.1007/s40572-023-00414-7 PMC11016309

[51] Hart JE, Chiuve SE, Laden F, Albert CM. Roadway proximity and risk of sudden cardiac death in women. Circulation. 2014;130(17):1474–82. 25332277 10.1161/CIRCULATIONAHA.114.011489 PMC4382912

[52] Kan H, Li T, Zhang Z. Air pollution and cardiovascular heath in a changing climate. Cardiol Plus. 2023;8(2):69–71.

[53] Yang T, Wu D, Zhang W, Cottrell RR, Rockett IR. Comparative stress levels among residents in three Chinese provincial capitals, 2001 and 2008. PLoS ONE. 2012;7(11):e48971. 23152832 10.1371/journal.pone.0048971 PMC3495856

[54] Manolis AA, Manolis TA, Melita H, Manolis AS. Takotsubo syndrome and sudden cardiac death. Angiology. 2023;74(2):105–28. 35668627 10.1177/00033197221105757

[55] Lou J, Chen H, Huang S, Chen P, Yu Y, Chen F. Update on risk factors and biomarkers of sudden unexplained cardiac death. J Forensic Leg Med. 2022;87:102332. 35272106 10.1016/j.jflm.2022.102332

[56] Shao F, Li H, Ma S, Li D, Li C. Outcomes of out-of-hospital cardiac arrest in Beijing: a 5-year cross-sectional study. BMJ Open. 2021;11(4):e041917. 33827829 10.1136/bmjopen-2020-041917 PMC8031015

[57] Zhao X, Zheng W, Ma Y, Hou Y, Zhu Y, Zheng J, et al. Epidemiology, process of care, and associated outcomes of pediatric out-of-hospital cardiac arrest in China: results from a prospective, multicenter, population-based registry. Crit Care Med. 2024;52(12):e604–15. 39637269 10.1097/CCM.0000000000006436

[58] Duber HC, Mcnellan CR, Wollum A, Phillips B, Allen K, Brown JC, et al. Public knowledge of cardiovascular disease and response to acute cardiac events in three cities in China and India. Heart. 2018;104(1):67–72. 28663360 10.1136/heartjnl-2017-311388

[59] Ibanez B, James S, Agewall S, Antunes MJ, Bucciarelli-Ducci C, Bueno H, et al. 2017 ESC Guidelines for the management of acute myocardial infarction in patients presenting with ST-segment elevation: the Task Force for the management of acute myocardial infarction in patients presenting with ST-segment elevation of the European Society of Cardiology (ESC). Eur Heart J. 2018;39(2):119–77. 28886621 10.1093/eurheartj/ehx393

[60] Velazquez EJ, Lee KL, Jones RH, Al-Khalidi HR, Hill JA, Panza JA, et al. Coronary-artery bypass surgery in patients with ischemic cardiomyopathy. N Engl J Med. 2016;374(16):1511–20. 27040723 10.1056/NEJMoa1602001 PMC4938005

[61] Schinkel AF, Vriesendorp PA, Sijbrands EJ, Jordaens LJ, Ten Cate FJ, Michels M. Outcome and complications after implantable cardioverter defibrillator therapy in hypertrophic cardiomyopathy: systematic review and meta-analysis. Circ Heart Fail. 2012;5(5):552–9. 22821634 10.1161/CIRCHEARTFAILURE.112.969626

[62] Tukker M, Schinkel AFL, Dereci A, Caliskan K. Clinical outcomes of implantable cardioverter-defibrillator therapy in noncompaction cardiomyopathy: a systematic review and meta-analysis. Heart Fail Rev. 2023;28(1):241–8. 35689132 10.1007/s10741-022-10250-w PMC9902401

[63] Zhang Y, Zhang J, Butler J, Yang X, Xie P, Guo D, et al. Contemporary epidemiology, management, and outcomes of patients hospitalized for heart failure in China: results from the China Heart Failure (China-HF) registry. J Card Fail. 2017;23(12):868–75. 29029965 10.1016/j.cardfail.2017.09.014

[64] Lv CZ, Zhang H, Chen S, Liu XR, Tian GG, Yan SJ. Consensus statement on layout and delivery of automatic external defibrillator in China. J Acute Dis. 2020;9(5):183–9.

[65] Shi HT, Ge JB. Improving public defibrillator use in China. Lancet. 2016;388(10050):1156–7. 27650087 10.1016/S0140-6736(16)31609-9

[66] Xie X, Zheng J, Zheng W, Pan C, Ma Y, Zhu Y, et al. Efforts to improve survival outcomes of out-of-hospital cardiac arrest in China: BASIC-OHCA. Circ Cardiovasc Qual Outcomes. 2023;16(2):e008856. 36503279 10.1161/CIRCOUTCOMES.121.008856

[67] Wang C, Zheng W, Zheng J, Shao F, Zhu Y, Li C, et al. A national effort to improve outcomes for in-hospital cardiac arrest in China: the BASeline investigation of cardiac arrest (BASIC-IHCA). Resusc Plus. 2022;11:100259. 35782311 10.1016/j.resplu.2022.100259 PMC9240856

