# Supplementary material for: Sudden cardiac arrest mortality in China: temporal trends and risk factors
Source: Mil Med Res. 2025 Aug 15;12:49. doi: 10.1186/s40779-025-00639-7 (PMC12355832; doi:10.1186/s40779-025-00639-7)
Supplement: Supplementary file 1 — Additional file 1. Materials and methods. Table S1 Standard certificate of death in China_English edition. Table S2 Codes used for screening in International Statistical Classification of Diseases and Related Health Problems 10th Revision ICD-10)-2015-World Health Organization version. Table S3 Average annual percent change (AAPC) of sudden cardiac mortality by periods in China. Table S4 Average annual percent change (AAPC) of sudden cardiac mortality stratified by sociodemographic factors in China. Table S5 Comparison of current status of trends, outcomes, and prevention practices for sudden cardiac arrest. Fig. S1 Flowchart for screening population for sudden cardiac death in China from 2013 to 2021 [file 40779_2025_639_MOESM1_ESM.pdf]

Materials and methods

Data source

The surveillance system has undergone 3 major improvements since 1978. First, it was expanded from 145 to 161 points in 2004 – 2006, yielding coverage of about 73 million residents. Second, a web-based approach was introduced to report deaths in 2008, a development that greatly improved the timeliness of data reporting. Third, the National Health and Family Planning Commission combined the vital registration system and disease surveillance points system in 2013 to create an integrated National Mortality Surveillance System (NMSS) [1,2]. The NMSS covers 605 surveillance points in 31 provincial-level administrative divisions in mainland China, accounting for 24.3% of the country's population with national and provincial representativeness [3]. For deaths that occurred in hospitals, doctors certified the cause of death and trained coders determined the underlying cause of death by applying the rules of the International Classification of Diseases, 10th Revision (ICD-10). For deaths occurring outside hospital, village health workers or community hospital staff did a verbal autopsy from which doctors in these hospitals determined the underlying cause of death. Since 2008, information on individual deaths in all population catchment areas has been reported in real time using an Internet-based reporting system. Information about causes of death in the certificate of death reported to the NMSS includes the immediate and intermediate causes of death and all other significant conditions contributing to the death. In this system, information on each death is systematically validated by local including county, prefecture, and provincial level Centers for Disease Control and Prevention (CDCs), which also check the completeness, coding, and internal logic of the items reported on death certificates. Causes-of-death are subsequently reported to the national CDC, where data are consolidated [4]. Information about causes of death in the certificate of death[5] (**Additional file 1: Table S1**) reported to the NMSS includes the immediate and intermediate cause of death (the final disease, injury, or complication directly leading to death), the antecedent causes (morbid conditions, if any, giving rise to the direct cause of death), and all other significant conditions contributing to the death but not related to the disease or condition causing it [6].

Table S1 Standard certificate of death in China_English edition

China standard certificate of death (translated by investigators)

<table>
<tr><td>Name</td><td></td><td>Gender</td><td></td><td>Ethnicity</td><td></td><td>Nation</td><td></td></tr>
<tr><td>Citizen ID type</td><td></td><td>Citizen ID number</td><td></td><td>Age</td><td></td><td>Marital status</td><td></td></tr>
<tr><td>Date of birth</td><td></td><td>Educational status</td><td></td><td>Occupation</td><td colspan="3"></td></tr>
<tr><td>Date of death</td><td></td><td>Place of death</td><td colspan="2">1) Hospital; 2) Prehospital; 3) Home; 4) Nursing home; 9) Other Places; 0) NA</td><td colspan="2">Pregenancy status</td><td></td></tr>
<tr><td>Working place</td><td></td><td>Registration address</td><td colspan="2"></td><td>Mailing address</td><td colspan="2"></td></tr>
<tr><td>Names of relatives</td><td></td><td>Tel</td><td colspan="2"></td><td>Address of relatives</td><td colspan="2"></td></tr>
<tr><td colspan="2"></td><td colspan="4">Cause of death (ICD code)</td><td colspan="2">Approximate interval: onset to death</td></tr>
<tr><td colspan="2">I. (a) Immediate cause</td><td colspan="4"></td><td colspan="2"></td></tr>
<tr><td colspan="2">(b) As a cause of (a)</td><td colspan="4"></td><td colspan="2"></td></tr>
<tr><td colspan="2">(c) As a cause of (b)</td><td colspan="4"></td><td colspan="2"></td></tr>
<tr><td colspan="2">(d) As a cause of (c)</td><td colspan="4"></td><td colspan="2"></td></tr>
<tr><td colspan="2">II. Underling cause (not directly related to death)</td><td colspan="4"></td><td colspan="2"></td></tr>
<tr><td>Health care institutions that determined the causes of deaths*</td><td colspan="4">(1) Tertiary care. (2) Secondary care. (3) Community care. (4) Primary care in village. (9) Other health care institution. (0) Home care</td><td>Strongest evidence of diagnosis*</td><td colspan="2">(1) Autopsy.
(2) Pathology.
(3) Surgery.
(4) Bedside clinical plus laboratory findings.
(5) Bedside clinical findings.
(6) Speculation.
(9) No available</td></tr>
<tr><td>Signature of physician</td><td></td><td colspan="3">Health care institution</td><td colspan="3">Date signed</td></tr>
<tr><td colspan="5">Underlying disease of death (filled by CDC staff)</td><td colspan="3">ICD code (filled by CDC staff)</td></tr>
</table>

*Serial numbers follow the non-consecutive sequence of the original Chinese table, which strictly conforms to the structure of the original 2013 form. *ICD* International Classification of Diseases, *CDC* Center for Disease Control and Prevention, *NA* not available

Table S2 Codes used for screening in International Statistical Classification of Diseases and Related Health Problems 10th Revision (ICD-10)-2015-World Health Organization version

ICD-10	ICD title
I46	Cardiac arrest
I49.0	Ventricular fibrillation and flutter
R95	Sudden infant death syndrome
R96	Other sudden death, cause unknown
Q20	Congenital malformations of cardiac chambers and connections
Q21	Congenital malformations of cardiac septa
Q22	Congenital malformations of pulmonary and tricuspid valves
Q23	Congenital malformations of aortic and mitral valves
Q24	Other congenital malformations of heart
Q25	Congenital malformations of great arteries
Q26	Congenital malformations of great veins
Q27	Other congenital malformations of peripheral vascular system
Q28	Other congenital malformations of circulatory system

ICD International Classification of Diseases

Table S3 Average annual percent change (AAPC) of sudden cardiac mortality by periods in China

Period	Crude mortality rate		Age-standardized mortality rate (ASMR)	
	**AAPC (95% CI)**	***P*-value**	**AAPC (95% CI)**	***P*-value**
Whole periods	10.88 (8.63 – 13.18)	< 0.001	7.12 (5.90 – 8.34)	< 0.001
Pre-COVID-19 period (2013 – 2019)	11.64 (9.79 – 14.48)	< 0.001	8.71 (7.73 – 10.08)	< 0.001
COVID-19 period (2020 – 2021)	8.64 (–1.30 to 13.38)	0.09	2.51 (–3.44 to 5.70)	0.22

Table S4 Average annual percent change (AAPC) of sudden cardiac mortality stratified by sociodemographic factors in China

Variables	AAPC	95% CI
**Age**		
0 – 14 years group	0.63	–6.48 to –8.29
15 – 34 years group	7.73	6.21 – 9.27
35 – 64 years group	8.87	6.41 – 11.38
≥ 65 years group	6.80	4.99 – 8.64
**Gender**		
Male	7.42	5.59 – 9.29
Female	6.30	3.59 – 9.07
**Marital status**		
Married	5.32	3.47 – 7.21
Unmarried	11.45	9.83 – 13.10
Widowed	11.29	5.49 – 17.40
Divorced	12.21	7.85 – 16.75
**Location**		
Urban	6.45	3.80 – 9.16
Rural	7.39	6.37 – 8.41
**Region**		
Eastern	7.47	5.40 – 9.57
Middle	5.80	4.11 – 7.52
Western	8.53	6.57 – 10.52

Table S5 Comparison of current status of trends, outcomes, and prevention practices for sudden cardiac arrest

Practices	China	Developed countries	Suggestions	Data source
Trends of SCD incidence	During 2013 – 2021, SCD increased markedly	No significant variability in the yearly incidence rates in Europe	-	[7]
Outcomes of SCA	Survival rate below 2% for adults	Survival to discharge rates are 2% in Asia, 9% in Europe, 6% in North America, and 11% in Australia	-	[8]
ICDs	In 2013: 1.4/1,000,000<br>Low rate of ICD utilization	In 2009, 434/1,000,000 in the United States		[9,10]
AEDs	Low rate of AED use (< 0.1%)<br>Poor public accessibility (< 1/1,000,000), awareness, knowledge, and user skills of AED	Public accessibility: 317/1,000,000 population in the United States	Public accessibility: (100 – 200)/100,000 population<br>Improve training about information and use of AED	[11-13]
CPR	Low implementation rate for bystander CPR, only 4.5% in 8 large and medium-sized cities	Implementation rate for bystander CPR is 46.1% in the United States, and 43.8% in Japan	"Platinum 10 min": timeliness is the key to successful CPR	[14-17]
Cardiopulmonary resuscitation training	Prevalent training rate in China is less than 1%<br>177 training centers<br>No detailed information about training quality	Prevalent training rate is 33% in the United States, and 40% in France;<br>72.1% agencies in the US provide ≥ once a year training for the use of mechanical CPR devices	More training at a higher standard	[14,18]
BLS training	The survey in developed cities: 20 – 48%	Global valid training: 10.02%; ever trained: 40.57%	Need for localization and co-construction	[19,20]

"-" indicates no data. *SCD* sudden cardiac death, *SCA* sudden cardiac arrest, *ICD* implantable cardioverter defibrillator, *AED* automated external defibrillator, *CPR* cardiopulmonary resuscitation, *BLS* basic life support

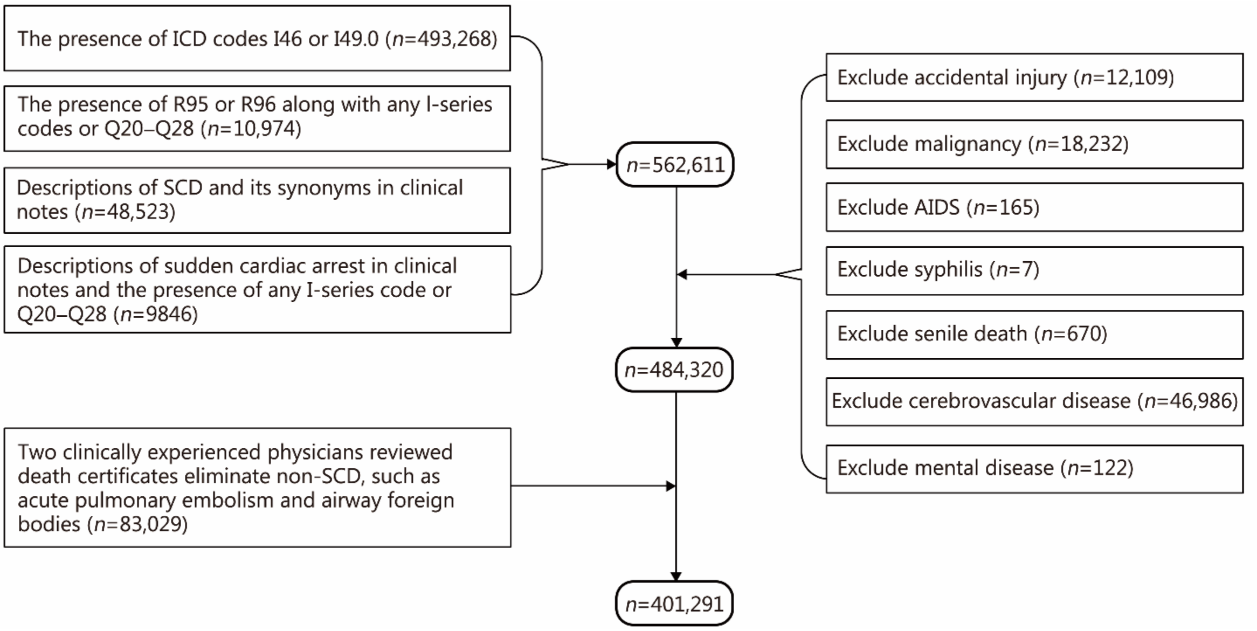

Fig. S1 Flowchart for screening population for sudden cardiac death in China from 2013 to 2021. ICD implantable cardioverter defibrillator, SCD sudden cardiac death, AIDS acquired immunodeficiency syndrome

References

1. Liu S, Wu X, Lopez AD, Wang L, Cai Y, Page A, et al. An integrated national mortality surveillance system for death registration and mortality surveillance, China. Bull World Health Organ. 2016;94(1):46-57.
2. Wang L, Wu Y, Yin P, Cheng P, Liu Y, Schwebel DC, et al. Poisoning deaths in China, 2006-2016. Bull World Health Organ. 2018;96(5):314-26a.
3. Qi J, Li M, Wang L, Hu Y, Liu W, Long Z, et al. National and subnational trends in cancer burden in China, 2005–20: an analysis of national mortality surveillance data. Lancet Public Health. 2023;8(12):e943-e55.
4. Weng L, Xu Y, Yin P, Wang Y, Chen Y, Liu W, et al. National incidence and mortality of hospitalized sepsis in China. Critical Care. 2023;27(1).
5. Chinese Center for Disease Control and Prevention. China Cause of Death Surveillance Dataset 2013. Beijing, China: Science Popularization Press; 2013.
6. Strengthening civil registration and vital statistics for births, deaths and causes of death: resource kit. http://apps.who.int/iris/bitstream/10665/78917/1/9789241504591_eng.pdf. Accessed 19 Feb 2018.
7. Schwaiger D, Krösbacher A, Eckhardt C, Schausberger L, Baubin M, Rajsic S. Out-of-hospital cardiac arrest: a 10-year analysis of survival and neurological outcomes. Heart Lung. 2025;73:1-8.
8. Berdowski J, Berg RA, Tijssen JG, Koster RW. Global incidences of out-of-hospital cardiac arrest and survival rates: systematic review of 67 prospective studies. Resuscitation. 2010;81(11):1479-87.
9. Mond HG, Proclemer A. The 11th world survey of cardiac pacing and implantable cardioverter-defibrillators: calendar year 2009--a World Society of Arrhythmia's project. Pacing Clin Electrophysiol. 2011;34(8):1013-27.
10. Zhang S. Sudden cardiac death in China: current status and future perspectives. Europace. 2015;17 Suppl 2:ii14-8.
11. Zhang L, Li B, Zhao X, Zhang Y, Deng Y, Zhao A, et al. Public access of automated external defibrillators in a metropolitan city of China. Resuscitation. 2019;140:120-6.
12. Rea T, Blackwood J, Damon S, Phelps R, Eisenberg M. A link between emergency dispatch and public access AEDs: potential implications for early defibrillation. Resuscitation. 2011;82(8):995-8.
13. Zheng J, Lv C, Zheng W, Zhang G, Tan H, Ma Y, et al. Incidence, process of care, and outcomes of out-of-hospital cardiac arrest in China: a prospective study of the BASIC-OHCA registry. Lancet Public Health. 2023;8(12):e923-e32.
14. Xu F, Zhang Y, Chen Y. Cardiopulmonary resuscitation training in China: current situation and future development. JAMA Cardiol. 2017;2(5):469-70.

15. Gu XM, Li ZH, He ZJ, Zhao ZW, Liu SQ. A meta-analysis of the success rates of heartbeat restoration within the platinum 10 min among outpatients suffering from sudden cardiac arrest in China. Mil Med Res. 2016;3:6.
16. Shao F, Li CS, Liang LR, Li D, Ma SK. Outcome of out-of-hospital cardiac arrests in Beijing, China. Resuscitation. 2014;85(11):1411-7.
17. Kitamura T, Kiyohara K, Sakai T, Matsuyama T, Hatakeyama T, Shimamoto T, et al. Public-access defibrillation and out-of-hospital cardiac arrest in Japan. N Engl J Med. 2016;375(17):1649-59.
18. Girotra S, Dukes KC, Sperling J, Kennedy K, Del Rios M, Crowe R, et al. Emergency medical service agency practices and cardiac arrest survival. JAMA Cardiol. 2024;9(8):683-91.
19. Ng TP, Eng SW, Ting JXR, Bok C, Tay GYH, Kong SYJ, et al. Global prevalence of basic life support training: a systematic review and meta-analysis. Resuscitation. 2023;186:109771.
20. Dong X, Zhang L, Wang Z, Zheng ZJ. Implementation of basic life support education for the lay public in China: barriers, enablers, and possible solutions. Front Public Health. 2024;12:1390819.
